# *ATP1A1 de novo* Mutation-Related Disorders: Clinical and Genetic Features

**DOI:** 10.3389/fped.2021.657256

**Published:** 2021-04-21

**Authors:** Zehong Lin, Jinliang Li, Taoyun Ji, Ye Wu, Kai Gao, Yuwu Jiang

**Affiliations:** Department of Pediatrics, Peking University First Hospital, Beijing, China

**Keywords:** *ATP1A1* gene, developmental delay, epilepsy, hypomagnesemia, genotype-phenotype correlations

## Abstract

**Background:** *ATP1A1* encodes an α1 isoform of Na^+^/K^+^-ATPase, which is expressed abundantly in kidneys and central nervous system. *ATP1A1* variants may cause Na^+^/K^+^-ATPase loss of function and lead to a wide spectrum of phenotypes. This study aims to summarize the clinical and genetic features of *ATP1A1 de novo* mutation-related disorders and explore the potential correlations between phenotypes and genotypes.

**Methods:** We analyzed two new cases harboring novel *de novo ATP1A1* variants and reviewed all reported cases.

**Results:** Both our probands had developmental delay, patient 1 accompanied with sleep disorders, irritability, and patient 2 with refractory seizures. They each had a novel *de novo* heterozygous missense variant, c.2797G>A[p.Asp933Asn] (NM_000701) and c.2590G>A[p.Gly864Arg] (NM_000701) respectively. Four patients with *de novo ATP1A1* variants have been reported in two previous papers. Among them, three patients had refractory seizures and one patient had complex hereditary spastic paraplegia (HSP). Therefore, all six patients had developmental delay, and four of them had epilepsy. All variants located in the transmembrane regions M3, M4, M7, and M8 of ATP1A1 protein. Four patients with mutations in M3 and M7 had more severe phenotypes, including developmental delay and epileptic encephalopathy, three of them with hypomagnesemia, whereas two patients with mutations in M4 and M8 had milder phenotypes, only with mild developmental delay, without seizures or hypomagnesemia. Correcting hypomagnesemia had not controlled those seizures.

**Conclusions:** Two novel *de novo ATP1A1* variants identified in two patients here enriched the genotypic and phenotypic spectrum of *ATP1A1* mutation-related disorder. Our findings suggest that hypomagnesemia in this disorder might relate to more severe phenotype and indicate more severe Na^+^/K^+^-ATPase dysfunction. Variations in M3 and M7 transmembrane regions were related to more severe phenotype than those in M4 and M8, which suggested that variations in M3 and M7 might cause more severe *ATP1A1* functional defect.

## Introduction

Na^+^/K^+^-ATPase is an integral membrane protein of a heterodimeric enzyme containing four isoforms of an α subunit (α1–α4) and three isoforms of a β subunit (β1–β3) (1). It can extrude three Na^+^ ions out of the cell and import two K^+^ ions into the cell using energy from the hydrolysis of one ATP in each pump cycle (2). It plays an essential role in maintaining the sodium and potassium ion concentration gradients at the plasma membrane of a cell, which are necessary for osmotic regulation, sodium-coupled transport, and the electrical excitability of nerves and muscles (1, 3).

*ATP1A1* encodes a transmembrane protein of 1,023 amino acids which is an α1 isoform of Na^+^/K^+^-ATPase. It locates in the human chromosome 1 region 1p2l → cen and practically ubiquitously expressed in body, especially in the kidneys and central nervous system (CNS) (1, 4). *ATP1A1* protein plays an important role in cation transport and responsible for establishing and maintaining the ion electrochemical gradients of Na^+^/K^+^ across the cell membrane. *ATP1A1* variants may cause Na^+^/K^+^-ATPase loss of function and abnormal cation permeability, leading to membrane depolarization and impairment of the activity of the physiological pump and thus to diseases (5). Previous studies have reported a range of diseases related to *ATP1A1* mutations, including renal hypomagnesemia, refractory epilepsy, intellectual disability, Charcot-Marie-Tooth disease (CMT), HSP, and aldosterone adenoma (3, 6–12). However, the phenotypes related to *ATP1A1 de novo* mutations were different from those with inherited mutations and have not been well-analyzed.

This study aimed to summarize the clinical and genetic features of *ATP1A1 de novo* mutation-related disorders and reveal the potential correlations between phenotypes and genotypes, including the two new cases we diagnosed and four previously reported cases.

## Patients and Methods

### Patients

We evaluated two Chinese patients harboring *de novo ATP1A1* pathogenic variants. Their clinical manifestations, serum Mg^+^ concentrations, brain magnetic resonance imaging (MRI), electroencephalogram (EEG), and *ATP1A1* variants were analyzed. Furthermore, all four previously reported cases with *de novo ATP1A1* mutations were also analyzed together in this study.

### Variation Analysis

Five milliliters of peripheral venous blood from each of the probands and their parents was collected, and genomic DNA was extracted for whole exome sequencing (WES) of family trios. The *in silico* tools PolyPhen-2, SIFT, and Mutation Taster and American College of Medical Genetics and Genomics (ACMG) guidelines were used to assess the pathogenicity of the two candidate variants (13).

## Results

### Clinical Features

Patient 1 was a girl aged 9 years and 5 months, with a *de novo ATP1A1* c.2797G>A [p.Asp933Asn] variant. Her motor development was normal. Her Wechsler Intelligence Scale for Children showed a Full Scale IQ (FSIQ) score of 71 at 7.5 years old, in the borderline range. She had poor executive functioning, and her learning ability, logical ability, and memory were significantly inferior to those of her peers. She began to experience sleep disorders when she was 2 years old, which manifested as waking up suddenly and crying after 3–4 h of sleep every night and the sleep disorder has relieved since her 8 years of age. In addition, she had poor emotional control, as easy to emotional collapse and cry constantly, but no epilepsy. Her brain MRI and serum Mg^2+^ concentrations were normal. Her birth history and family history were normal.

**Figure 1:**
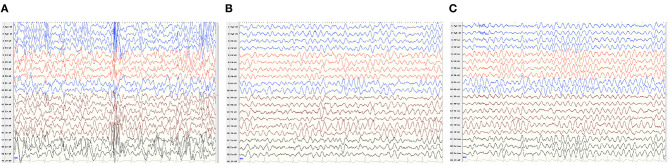
V-EEG: Widespread slow waves and spike slow waves during sleep and 3–4 Hz slow waves, especially in para-midline and midline regions, during waking, and sleeping periods. **(A–C)** EEG features described as the caption of Patient 2.

Patient 2 was a boy aged 2 years and 10 months, with a *de novo ATP1A1* c.2590 G>A[p.Gly864Arg] variant. He had severe developmental delay, manifesting as sitting independently but unstably, he couldn't say any words and only made a babbling sound by the time of our follow-up. He began to develop epileptic seizures 5 months after birth, characterized by a tonic seizure in one limb and, later, evolving into generalized tonic–clonic seizures. EEG showed widespread slow waves and spike slow waves during sleep and wide 3–4 Hz slow waves, especially in para-midline and midline regions, during waking and sleeping stages (Figure 1). He was diagnosed as developmental and epileptic encephalopathy. His brain MRI and serum Mg^2+^ concentrations were normal. The child has taken a total of three antiepileptic drugs including diazepam, levetiracetam, and topiramate, the latter two are currently used, but the seizures have not been controlled. He was born at 36 6/7 weeks of gestation with normal delivery. His family history was unremarkable.

### Molecular Findings

**Figure 2:**
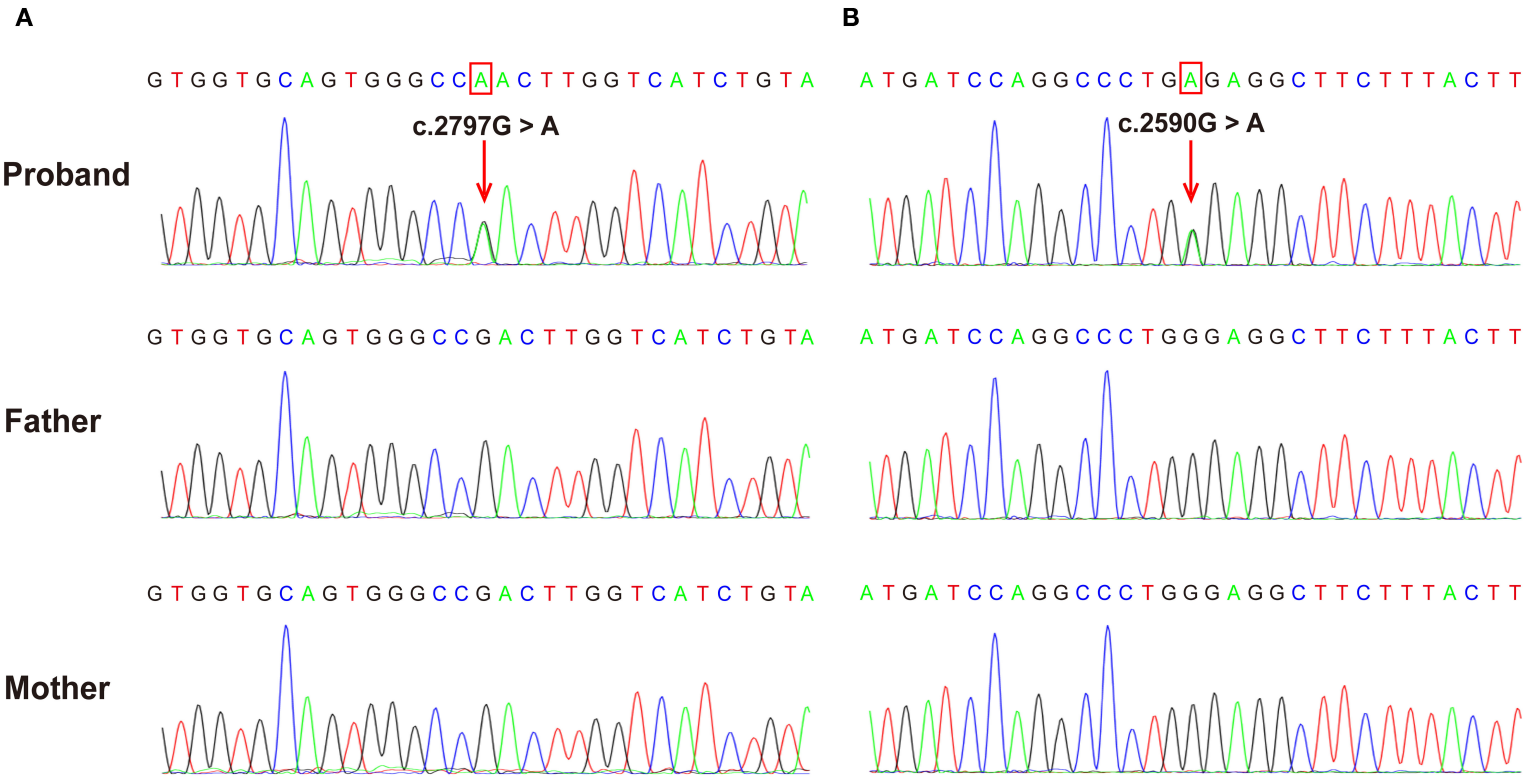
The *de novo ATP1A1* variants were confirmed using Sanger sequencing. **(A)** c.2797G>A variant in case 1 resulting in a p.Asp933Asn substitution. **(B)** c.2590G>A variant in case 2 resulting in a p.Gly864Arg substitution. Verified as *de novo* variants using Trio-WES and Sanger sequencing.

Both patients were identified *de novo* variants of *ATP1A1*. Patient 1 was found to have c.2797G>A[p.Asp933Asn] and patient 2 was found to have c.2590 G>A[p.Gly864Arg]; variants were *de novo* in both cases (Figure 1). Sequence alignment among multiple species suggested that p.Asp933Asn and p.Gly864Arg were located in highly conserved sites (Figure 2). Variants were predicted to be pathogenic by multiple bioinformatic tools (e.g., PolyPhen-2, SIFT, and Mutant Taster. In addition, they were not included in genomAD, ExAC, and 1000 genomes. According to ACMG guidelines, we confirmed that both variants are likely pathogenic (see Table 1 for details). Furthermore, we did not find other pathogenic variants of genes known to be associated with CNS-related diseases in the two probands using Trio-WES.

**Table 1:** Pathogenic prediction in *ATP1A1* variants.

**Case**	**Mutation**	**Variant origin**	**MAF**			**Polyphen-2**	**SIFT**	**Mutation taster**	**ACMG guideline**	**Category**
			**genom-AD**	**ExAC**	**1000 genomes**					
1	*ATP1A1*	p.Asp933Asn	–	–	–	PD	D	DC	PS2+PM1+PM2+PP3+PP2	LP
2	*ATP1A1*	p.Gly864Arg	–	–	–	PD	D	DC	PS2+PM1+PM2+PP3+PP2	LP

*PD, Probably Damaging; D, Damaging; DC, disease causing; LP, Likely Pathogenic*.

### Literature Review Findings

We searched PubMed with the keywords “*ATP1A1*” and “*de novo*.” A total of four children with *de novo* variants in *ATP1A1* were identified.

Schlingmann et al. (9) first described three patients harboring the *de novo* variations c.905T>C[p.Leu302Arg], c.907G>C[p.Gly303Arg], and c.2576T>G[p.Met859Arg] (NM_000701) in *ATP1A1*, involving renal hypomagnesemia, refractory epilepsy, and intellectual disability[HOMGSMR2, MIM618314] (Table 2, cases 3, 4, and 5). The onset ages of the three patients ranged from 6 days to 6 months. The first symptoms were generalized seizures and severe hypomagnesemia. All patients had received antiepileptic drugs and magnesium supplementation, but seizures continued. Moreover, all three patients involved severe generalized developmental delay. Among them, two patients (Table 2, cases 4 and 5) also showed additional clinical features consistent with autism spectrum disorders. Two patients underwent brain MRI, which both showed cerebral atrophy. None of the children's parents had any related clinical manifestations or hypomagnesemia.

**Table 2:** Clinical features of patients with *ATP1A1* mutations.

**Case**	**Origin**	**Age at Manifestation**	**Gender**	**Clinical symptom**	**Developmental delay (DD)**	**Hypomagnesemia**	**Outcome**	**Mutation (*de novo*) (NM_000701)**	**Genetic testing**
1^a^	Chinese	2 y	F	Sleep disorder, irritability	Borderline intellectual Functioning (WISC-IV IQ Scores 71)	–	The sleep disorder self-relieved at 8 y, motor coordination is a little behind	p. Asp933Asn	Trio-WES
2^a^	Chinese	5 mo	M	Focal seizure	Sever global delay	–	Seizures frequency decrease	p. Gly864Arg	Trio-WES
3 (9)	European descent	6 mo	F	Generalized seizures, hyperactive behavior	Global developmental delay	+	Repeated status epilepticus	p. Leu302Arg	Proband-WES+Parents validated
4 (9)	European Descent	2 mo	F	Generalized seizures, suspected autism Spectrum disorder	Global developmental delay	+	Monthly seizures	p. Gly303Arg	Proband-conventional Sanger Sequencing +Parents Validated
5 (9)	First Nations Canadian	6 d	M	Generalized seizures, speech delay, diagnosis of severe autism, self-biting behavior	Global developmental delay	+	Frequent seizures, repeated status epilepticus	p. Met859Arg	Trio-WES
6 (10)	Romanian	1 y	M	Irritability, attention deficit, hyperactivity, and temper tantrum were noticed sleep disorder	Spastic gait with pyramidal signs at the lower limbs, language was limited to 3–5 simple words at 2.5 y, moderate severity DD (3 y10mo: GMDS-III DQ <50, VABS-II adaptive composite score 42)	–	NA	p. Leu337Pro	Trio-WES

a *Our case*.

*NA, not applicable; WISC-IV, Wechsler Intelligence Scale for Children, fourth edition; IQ, intelligence quotient; GMDS-III, Griffiths Mental Development Scales, third version; DQ, Developmental Quotient; VABS-II, Vineland Adaptive Behavior Scales, second version; WES, whole exon sequencing*.

Stregapede et al. (10) reported a *de novo ATP1A1* mutation-related HSP, with the c.1010 T>C[p.Leu337Pro] variant (NM_000701) (Table 2, case 6). The patient was 4 years and 6 months old. He did not have epilepsy, but exhibited motor developmental delay in his first year of life. He could sit independently at the age of 12 months, walk at 18 months with an abnormal gait and frequent falls, and speak 3–5 simple words at 2 years and 6 months; his eye contact was normal. In addition, attention-deficit hyperactivity disorder, temper tantrums, and sleep disorders were identified in the patient. Both MRI (brain and spine) and serum Mg^2+^ concentrations were normal. Neurologic examination revealed pyramidal signs at the lower limbs. Motor evoked potential revealed that the central conduction time at the lower limbs was increased. EEG and nerve conduction study of the upper and lower limbs were inconclusive. Neuropsychological assessments suggested moderate to severe developmental delay at 3 years and 10 months (Griffiths Mental Development Scales, third version: developmental quotient <50 and developmental age equivalent to 29 months; Vineland Adaptive Behavior Scales, second version: adaptive composite score 42).

### Summary

**Figure 3:**
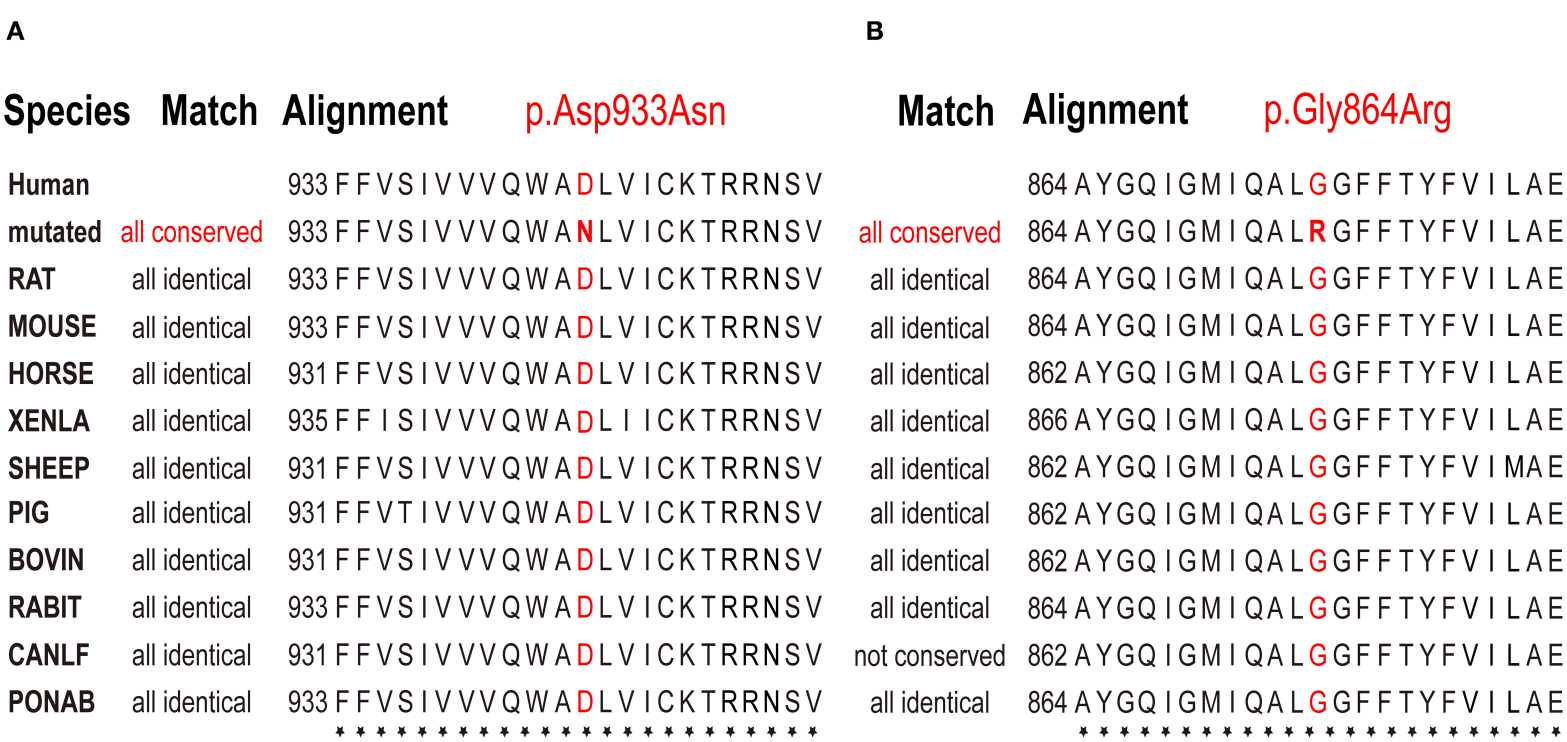
Multiple species sequence alignment. Conservation analysis of amino acid sequences on p.Asp933Asn and p.Asp933Asn variant sites demonstrated that they were located on highly conserved sites. The bold red letters mean the amino acids that represent the same location in different species are all the same, indicated that they are highly conserved. **(A)** p.Asp933Asn variant in case 1, **(B)** p.Gly864Arg variant in case 2.

In brief, including the two cases we reported here, there have been six patients with *de novo ATP1A1* mutations identified to date. The pathogenic *ATP1A1* variants in these patients were p.Asp933Asn, p.Gly864Arg, p.Leu302Arg, p.Gly303Arg, p.Met859Arg, and p.Leu337Pro (Figure 3). All patients had different degrees of developmental delay, and four had epilepsy. Of the four patients with epilepsy, three had generalized epileptic seizures and hypomagnesemia, and one patient had focal epileptic seizure without hypomagnesemia. Antiepileptic medicine or magnesium supplementation cannot control epileptic seizures in all these patients.

## Discussion

**Figure 4:**
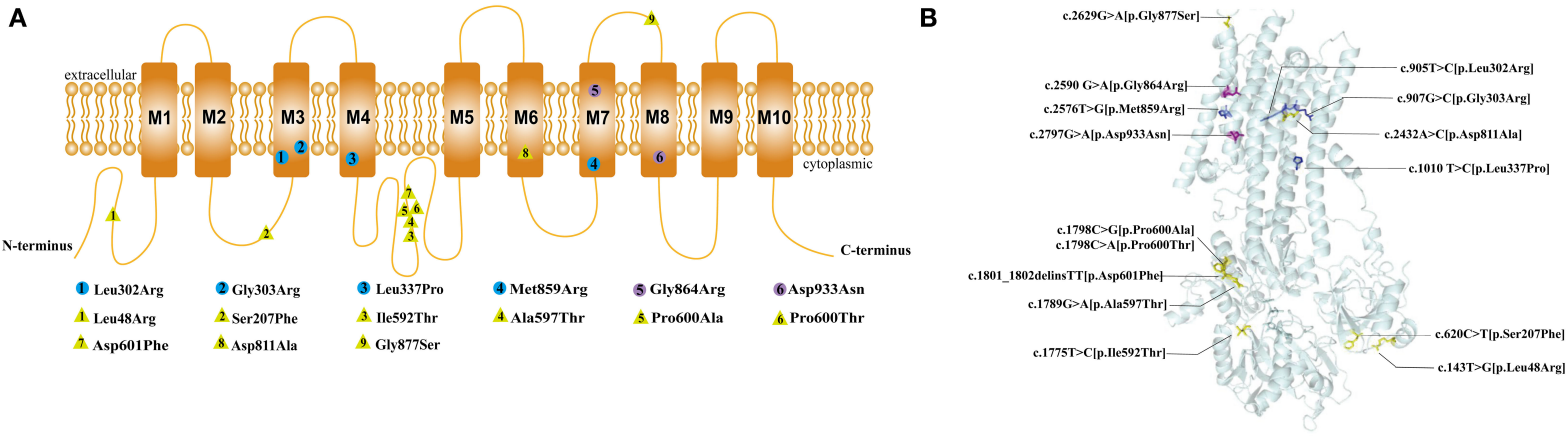
Location of the pathogenic variants in ATP1A1 protein. **(A)** 2D structure: The circles represent the *de novo* variants, with purple filled representing the novel variants that we found and blue filled representing the previously reported variants. The yellow triangles represent inherited variants associated with CMT previously reported. **(B)** 3Dstructure: Location of all the pathogenic variants in ATP1A1 protein. All the *de novo* mutations were concentrated in the transmembrane region, whereas 88.9% (8/9) of inherited variants concentrated in the topological regions, and 11.1% (1/9) locate in the transmembrane regions.

The *ATP1A1* protein is composed of ten transmembrane helices and three cytoplasmic domains: A (actuator), N (nucleotide-binding), and P (phosphorylation) (14). All *de novo ATP1A1* variants located in the regions encoding transmembrane regions M3, M4, M7, and M8 of the ATP1A1 protein (Figure 4), which suggests that these regions may be susceptible to *de novo ATP1A1* mutation. Interestingly, the amino acid residues in M3 and M7 (two cases each: M3: p.Leu302Arg, p.Gly303Arg; M7: p.Gly864Arg, p.Met859Arg) were all changed to arginine. If a mutation introduces an arginine into the transmembrane region, this positively charged residue may interfere with the negatively charged ion binding sites of the pump, and generally, a charged residue is unfavorable in the transmembrane region. Such mutations are therefore more likely to result in pump dysfunction, either because they directly disrupt binding and transport of sodium and/or potassium, or because they disturb the overall structure.

To date, the phenotype spectrum of *de novo ATP1A1* pathogenic variants has included neurodevelopmental disorders (including developmental delay, autism spectrum disorders, and epilepsy), emotional disorders, sleep disorders, and HSP. It is suggested that *ATP1A1* mutation-related disorders may have a wide spectrum of phenotypes, which may be related to the extensive expression and important functions of *ATP1A1* in the nervous system (1, 4, 5). Among neurodevelopmental disorders, four patients (age from 2 months to 10 years) had generalized developmental delay, and one patient with HSP had significant language retardation. Four patients (4/6) were diagnosed with epilepsy, including three patients with generalized epileptic seizure and one patient with focal seizure; the generalized seizure patients featured hypomagnesemia, while the focal seizure patient did not. Seizures were not controlled after combined treatments of several antiepileptic drugs and magnesium supplementation. EEG features of *ATP1A1* mutation related epilepsy were not described in previous reports. Patient 2 exhibited focal epileptic seizure with severe developmental delay, and MRI showed normal brain structure. His EEG showed the features of genetic developmental and epileptic encephalopathy.

The mechanism of hypomagnesemia caused by *ATP1A1* variants is associated with dysfunction of renal tubular Na^+^/K^+^-ATPase. The Na^+^/K^+^-ATPase expressed on the outside of the renal tubule basement membrane establishes a favorable electrochemical gradient for the influx of cations through the magnesium ion channel and provides an exit mechanism for reabsorbed sodium ions. The distal convoluted tubule mediates active transcellular magnesium transport and has the highest Na^+^/K^+^-ATPase density and activity; *ATP1A1* encodes the most prevalent form of Na^+^/K^+^-ATPase in the distal convoluted tubule (9). Therefore, *ATP1A1* variants may lead to Na^+^ and K^+^ transport dysfunction and interfere with the active reabsorption of magnesium ions, resulting in hypomagnesemia. In our cases, we found that seizures were not controlled after magnesium supplementation. This supported that epilepsy in these patients might be a direct result of neuronal energy dysfunction caused by Na^+^/K^+^-ATPase dysfunction in the CNS rather than a secondary feature of hypomagnesemia (9).

Hypomagnesemia in the patients with *ATP1A1* mutations may indicate more severe Na^+^/K^+^-ATPase dysfunction, because the epileptic phenotype occurs more often in individuals with hypomagnesemia. All three patients with hypomagnesemia had refractory epilepsy, whereas among the three patients without hypomagnesemia, only one had refractory focal epilepsy, and the other two did not have seizures. Four patients with epilepsy had significant language delay. Among them, three patients (age from 4 to 10 years) could only speak a single word; one patient, aged 2 years and 10 months, could only make a babbling sound. The two patients without epilepsy had less developmental delay, one of them had mild language delay, and the other had normal language development.

Four patients with mutations in M3 and M7 (two cases each) had more severe phenotypes, which manifested as developmental delay and epileptic encephalopathy; three of these patients featured hypomagnesemia. However, two other patients with mutations in M4 and M8 had less severe phenotypes, which mainly manifested as mild developmental delay, sleep disorders, emotional disorders, HSP, and a lack of seizures or hypomagnesemia. This suggests that variations in M3 and M7 may have a greater detrimental impact on *ATP1A1* function and lead to more severe phenotypes.

In contrast to patients with *de novo* variants, patients with inherited variants are mainly presented CMT. *Eight* patients had familiar *ATP1A1* mutation-related autosomal dominant CMT, one of them inherited the mutation from his mother with mosaic mutation (3, 8). The inheritance mode of one case with CMT could not be confirmed due to lacking of the father's genetic test (3). The age of onset ranged from 8 to 50 years old, and the mutations mainly caused weakness and atrophy of the distal extremities and sensory disturbances rather than seizures. No information about the blood magnesium test were found in those previous reports. These mutations were mainly concentrated in the topological regions of ATP1A1 (88.9%, 8/9), and one (11.1%, 1/9) located at the transmembrane region, suggesting that topological regions may have a specificity for peripheral neuropathy. Sweadner et al. (15) reported that the putatively pathogenic mutations of *ATP1A1* found in CMT2 axonal sensorimotor neuropathy were mainly in the P domain, N domain, or extracellular surface which may affect only biosynthesis or activity. But the pathogenic mutations of *ATP1A1* found in patients with epilepsy were all in the transmembrane regions which significantly reduced the K+ affinity and even cooperativity of K+ binding (9). In the CNS, constant Na^+^/K^+^-ATPase activity is required for generating the resting membrane potential and buffering and clearance of extracellular K^+^ transients during neuronal activity. It suggested that the decreases in Na^+^/K^+^-ATPase activity from these mutations might cause more sever phenotypes.

In summary, two novel *de novo ATP1A1* variants were identified in two cases in this study, enriching the phenotypic spectrum of *ATP1A1* mutation-related genetic diseases. Together with four previously reported cases, we summarized the clinical characteristics of all six patients with *ATP1A1 de novo* mutation-related diseases. The most common phenotypes are developmental delay and refractory epilepsy. *ATP1A1* variants can cause dysfunction of renal tubular reabsorption, leading to hypomagnesemia, but seizures were not controlled by magnesium supplementation. Our findings suggest that seizures are not secondary to hypomagnesemia but rather caused directly by Na^+^/K^+^-ATPase dysfunction in the CNS. However, hypomagnesemia might indicate more severe Na^+^/K^+^-ATPase dysfunction, particularly in the CNS, because patients with hypomagnesemia are more likely to have epilepsy. Moreover, the transmembrane regions M3, M4, M7, and M8 might be susceptible to *de novo ATP1A1* mutations. The *ATP1A1* variations located in M3 and M7 might have a greater detrimental impact on *ATP1A1* function and lead to more severe phenotypes. However, the case number were too limited to clarify the complete features for this disease, and further large-sample studies are needed to confirm our findings.

## Data Availability Statement

The original contributions presented in the study are included in the article/supplementary material, further inquiries can be directed to the corresponding author/s.

## Ethics Statement

The studies involving human participants were reviewed and approved by Institutional Review Board of Peking University First Hospital. Written informed consent to participate in this study was provided by the participants' legal guardian/next of kin. Written informed consent was obtained from the individual(s), and minor(s)' legal guardian/next of kin, for the publication of any potentially identifiable images or data included in this article.

## Author Contributions

YJ and KG: study design, analysis, and revision of the manuscript. ZL: follow-up of patient's information and draft preparation. ZL, JL, TJ, YW, KG, and YJ: collection of clinical and WES data. All authors contributed to the article and approved the submitted version.

## Conflict of Interest

The authors declare that the research was conducted in the absence of any commercial or financial relationships that could be construed as a potential conflict of interest.
